# Tuberculous Spondylitis following Intravesical* Bacillus* Calmette-Guerin for Bladder Cancer

**DOI:** 10.1155/2016/6741284

**Published:** 2016-05-30

**Authors:** Masashi Miyazaki, Toyomi Yoshiiwa, Toshinobu Ishihara, Masanori Kawano, Hiroshi Tsumura

**Affiliations:** Department of Orthopaedic Surgery, Oita University, 1-1 Idaigaoka, Hasama-machi, Yufu-shi, Oita 879-5593, Japan

## Abstract

We present a rare case of tuberculous spondylitis following intravesical* Bacillus *Calmette-Guerin (BCG) therapy for bladder cancer. An 82-year-old man presented with low back pain. Past medical history revealed bladder cancer diagnosed and treated 16 months previously by intravesical BCG. Magnetic resonance imaging of the thoracic spine showed destruction of the T5 and T6 vertebrae and an epidural soft tissue mass with anterior dural sac compression. Due to the progression of vertebral destruction, posterior spinal segmental fusion was performed.* Mycobacterium bovis (M. bovis)* was identified using multiplex polymerase chain reaction of surgical tissue specimens. The patient was started on an antituberculosis treatment regimen including isoniazid, rifampicin, and ethambutol. After surgery, his back pain resolved completely. At the latest examination, the patient was pain-free with no functional limitations or recurrent infection in clinical or imaging findings. Patients undergoing BCG therapy should be monitored for possible hematogenous spread of mycobacteria to the spine for months or even years after treatment.

## 1. Introduction

*Bacillus* Calmette-Guerin (BCG), an attenuated derivative of the virulent strain of* Mycobacterium bovis (M. bovis)*, is widely used and effective in preventing superficial bladder cancer recurrence [1–3]. Serious complications are rare, and musculoskeletal complications are limited to a 0.5% incidence of arthralgia and migratory arthritis [4]. The incidence and type of complications depend on the particular strain, dose, and route of administration. BCG osteitis commonly involves the lower limbs, ribs, and sternum, but spinal involvements are extremely rare and, to the best of our knowledge, only 7 cases have been reported to date. We present a case of* M. bovis* tuberculosis spondylitis occurring 16 months after intravesical BCG therapy in an immunocompetent patient with bladder carcinoma.

## 2. Case Report

An 82-year-old man was admitted with a 2-month history of low back pain and no history of trauma, fever, night sweats, or cough. Past medical history revealed bladder cancer diagnosed 16 months previously and treated with 8 cycles of intravesical BCG.

**Figure 1:**
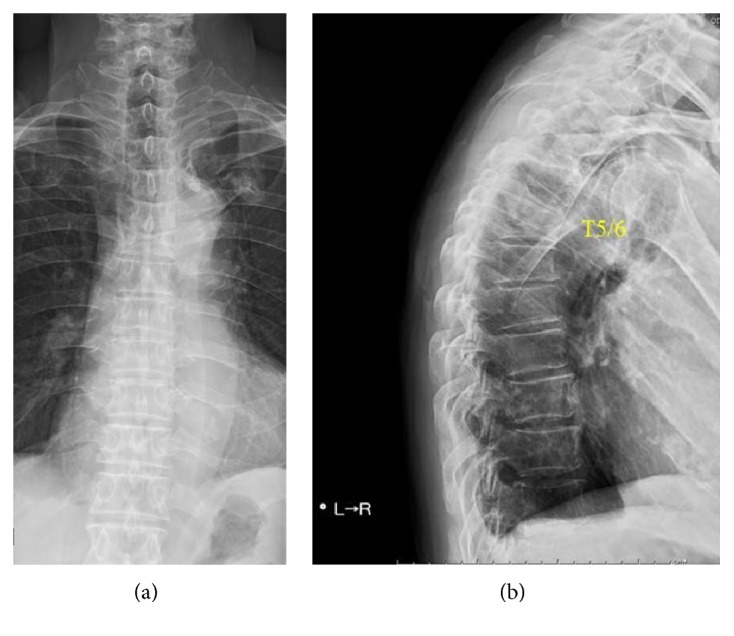
(a) Anteroposterior and (b) lateral radiographs of the thoracic spine show compression fractures of the T5 and T6 vertebrae.

**Figure 2:**
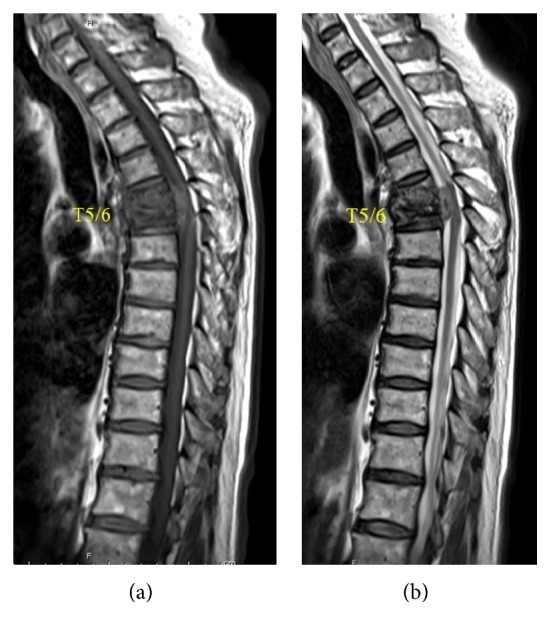
(a) Sagittal T1-weighted and (b) sagittal T2-weighted magnetic resonance imaging of the thoracic spine shows destruction of the T5 and T6 vertebrae and an epidural soft tissue mass with anterior dural sac compression.

The patient was admitted with back pain and tenderness on palpation and percussion over the spinous processes of the upper thoracic vertebrae. A lateral pelvic compression test and extreme range of motion of the hips and sacroiliac joints were painless. Deep tendon reflexes were normal. There was no motor weakness or paresthesia. Initial routine laboratory examination showed mild normochromic normocytic anemia, normal white blood cell count, an erythrocyte sedimentation rate of 33 mm/first hour, and a C-reactive protein of 0.75 mg/dL. The QuantiFERON-TB test result was negative. Plain radiographs showed T5 and T6 compression fractures (Figures 1(a) and 1(b)). A chest radiograph was unremarkable. Thoracic spine magnetic resonance imaging showed T5 and T6 destruction and an epidural soft tissue mass with anterior dural sac compression (Figures 2(a) and 2(b)).

**Figure 3:**
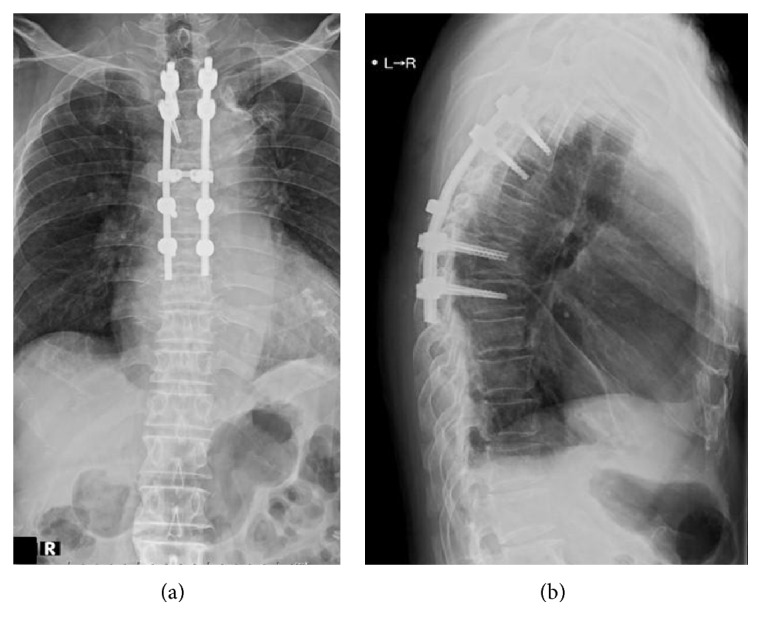
(a) Anteroposterior and (b) lateral radiographs show T3–8 posterior spinal segmental instrumentation and fusion.

**Figure 4:**
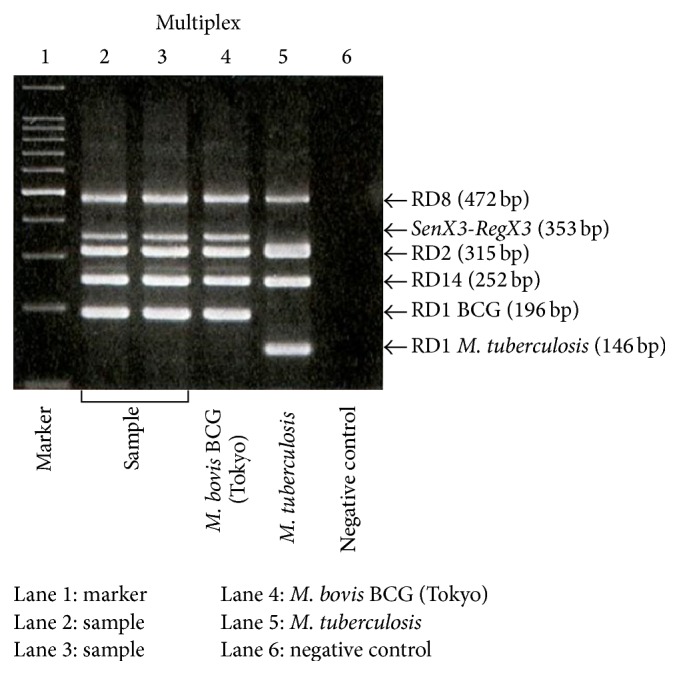
*M. bovis* was identified using multiplex polymerase chain reaction.

Computed tomography-guided needle biopsy was performed for suspicion of a tuberculous infection. Histological examination of the biopsy specimen revealed a granulomatous infection. Due to progressive vertebral destruction and severe low back pain, T3–8 posterior spinal segmental instrumentation and fusion was performed via a posterior approach (Figures 3(a) and 3(b)). Tissue specimens via pedicle were obtained intraoperatively.* M. bovis* was subsequently identified using multiplex polymerase chain reaction (Figure 4). The diagnosis of tuberculosis was confirmed and hematogenous dissemination to the thoracic spine following intravesical BCG therapy for bladder cancer was postulated. The patient was started on antituberculosis treatment including isoniazid, rifampicin, and ethambutol.

After surgery, the patient's back pain resolved completely. Antituberculous treatment was completed 6 months after diagnosis and surgical treatment. One year after surgery, the patient was pain-free with no functional limitations or recurrent infection.

## 3. Discussion

Intravesical BCG immunotherapy for treatment of superficial bladder cancer and in situ carcinomas has a low risk of adverse effects, most of which are localized and self-limited [5]. Although adverse effects such as cystitis, fever, hematuria, prostatitis, arthralgias, and reactive arthritis are relatively common, extravesical complications are rare [5]. Previous studies reported BCG spondylitis following intravesical BCG immunotherapy, but only 7 cases of tuberculous spondylitis related to intravesical BCG immunotherapy for bladder cancer have been reported [6–13].

There is no obvious evidence regarding the mechanism of systemic manifestations that are related to BCG immunotherapy or vaccination. The possibility of both dissemination of BCG infection and hypersensitivity reactions has been debated. However, positive cultures for* M. bovis* bacilli obtained from a distant site strongly suggest dissemination of BCG infection [6, 8]. Hematogenous spread to the anterior vertebral bodies of the spine where the arterial supply converges is a pathogenesis shared with other bacterial infections [14], predicting involvement of the anterior vertebral body adjacent to the endplate with spread to the contiguous vertebrae along ligamentous planes. Venous drainage may also contribute to infectious vertebral pathology [15]. The timeframe for BCG spondylitis onset varies considerably. In most cases, BCG spondylitis occurs soon after intravesical BCG therapy but has been reported 12 years later [13]. Therefore, patients having BCG therapy should be closely evaluated for hematogenous spread of mycobacteria to distant sites for months or even years after treatment.

Several risk factors are considered with an incidence of dissemination of viable* M. bovis* bacilli. These are a bladder epithelium injury, urethral injury during BCG instillation, deep bladder tumor resection, pelvic radiation, severe cystitis and bladder biopsy, or prostate resection [8, 16, 17]. Bacilli of BCG origin have appeared in bladder biopsy specimens and early-morning urine cultures for over a year after intravesical BCG therapy [17]. This may explain why patients are at risk of disseminated infection for months or even years after intravesical BCG therapy.

It is very important to reliably distinguish* M. bovis* BCG strains from* M. tuberculosis *to differentiate between BCG reactivation and reinfection with* M. tuberculosis*. Recently, rapid molecular diagnostic testing has been developed that differentiates* M. bovis* BCG from its parent* M. bovis* strain [18]. Other methods of identifying BCG strains include phage typing, high performance liquid chromatography, restriction fragment length polymorphism analysis, dot blot enzyme-linked immunosorbent assay (ELISA) testing, and PCR identification of* M. bovis* [7, 18].

The role of prophylactic antituberculous therapy accompanying intravesical BCG therapy is debated. Rawls et al. recommended 3 days of prophylactic isoniazid therapy beginning the morning before treatment [19]. However, Fishman et al. reported BCG spondylitis even with prophylactic isoniazid coverage [7]. A large prospective study is needed to assess whether prophylactic agents reduce intravesical BCG therapy complications.

Unlike* M. tuberculosis*, all strains of* M. bovis* are resistant to pyrazinamide. Hence, isoniazid and rifampicin, with or without ethambutol, should be used as first-line agents to treat* M. bovis* infections [12, 20]. In cases of disseminated systemic infection or specific organ involvement, triple-regimen antituberculous therapy is warranted. However, surgical intervention should be considered if a tuberculous spondylitis is resistant to antibiotics, or neurological symptoms or spinal instability appears [21]. For* M. bovis* tuberculous spondylitis, surgical intervention is usually necessary for spinal instability [6–10, 12, 13]. In our case, due to progressive vertebral destruction and severe low back pain, posterior spinal instrumentation and fusion was necessary.

In conclusion, we presented a rare case of intravesical BCG therapy complicated by* M. bovis* spondylitis. Predisposing factors to dissemination should be addressed to minimize the risk of hematogenous spread of live attenuated BCG to the spine. Furthermore, the timeframe for BCG spondylitis onset after intravesical BCG therapy is variable. Patients having BCG therapy should be monitored for possible hematogenous spread of mycobacteria, which can arise months or even years after therapy. If BCG spondylitis is suspected, diagnosis and treatment are needed to avoid unfavorable sequelae.
